# Update on functional analysis of long non-coding RNAs in common crops

**DOI:** 10.3389/fpls.2024.1389154

**Published:** 2024-05-30

**Authors:** Aijing Zhang, Wenxuan Pi, Yashuo Wang, Yuxin Li, Jiaxin Wang, Shuying Liu, Xiyan Cui, Huijing Liu, Dan Yao, Rengui Zhao

**Affiliations:** ^1^College of Life Science, Jilin Agricultural University, Changchun, Jilin, China; ^2^College of Agronomy, Jilin Agricultural University, Changchun, China

**Keywords:** lncRNAs, plant development, biological function, molecular mechanism, crops

## Abstract

With the rapid advances in next-generation sequencing technology, numerous non-protein-coding transcripts have been identified, including long noncoding RNAs (lncRNAs), which are functional RNAs comprising more than 200 nucleotides. Although lncRNA-mediated regulatory processes have been extensively investigated in animals, there has been considerably less research on plant lncRNAs. Nevertheless, multiple studies on major crops showed lncRNAs are involved in crucial processes, including growth and development, reproduction, and stress responses. This review summarizes the progress in the research on lncRNA roles in several major crops, presents key strategies for exploring lncRNAs in crops, and discusses current challenges and future prospects. The insights provided in this review will enhance our comprehension of lncRNA functions in crops, with potential implications for improving crop genetics and breeding.

## 1 Introduction

Noncoding RNAs (ncRNAs), which do not encode proteins and were originally considered to be “transcriptional noise,” account for most of the total RNA in cells (Nojima and Proudfoot, 2022). With the development and application of transcriptomic technology, the importance of an increasing number of ncRNAs for genomic organization and function has been revealed (Jha et al., 2023). In fact, ncRNAs have gradually become a major focus of life sciences research (Waititu et al., 2022; Cui, 2023). The two types of ncRNAs are distinguished by their mechanism of action. Specifically, housekeeping ncRNAs include transfer RNAs (tRNAs), small nuclear RNAs (snRNAs), small nucleolar RNAs (snoRNAs), and ribosomal RNAs (rRNAs), whereas regulatory ncRNAs include short interfering RNAs (siRNAs), microRNAs (miRNAs), PIWI-interacting RNAs (piRNAs), and long noncoding RNAs (lncRNAs) (Duan X. et al., 2020; Virciglio et al., 2021). Among these ncRNAs, lncRNAs affect gene expression through a wide range of mechanisms and are essential regulators of many important biological processes (Qin et al., 2017; Ahmed et al., 2020).

The first stage of research on lncRNAs was from 1980 to 2000, during which lncRNAs were first identified using traditional gene mapping methods, with *H19* being one of the first reported lncRNAs (Yoshimura et al., 2018; Yang et al., 2021). Additionally, *XIST*, the main regulator of X-chromosome inactivation, was also discovered in this period (Hierholzer et al., 2022). In the second stage, which involved a shift from the noncoding genome to the noncoding transcriptome, thousands of lncRNAs were identified in plants. In the third stage, microarrays, tiled arrays, and next-generation sequencing technologies were used to identify regulatory lncRNAs and clarify their involvement in many processes, such as development and pathogenesis, in numerous plant species (Jarroux et al., 2017; Wu et al., 2020). The increasing functional characterization of lncRNAs has been accompanied by an increase in the number of studies on lncRNAs over the last decade. The mechanisms of action of lncRNAs in animals have been extensively studied (Zhang et al., 2020; Zhang X. et al., 2023). Moreover, there has been a steady increase in the research on lncRNAs in both animals and plants over the years. The resulting published articles reflect the growing interest, funding, and research on crop lncRNAs. However, plant lncRNA studies lag behind those on animal lncRNAs, likely because of the delayed initiation of plant research. Nevertheless, lncRNAs in major crops, such as rice, maize, and cotton, have been identified and characterized. Technological advances may be exploited to further expand the research on plant lncRNAs. A comprehensive overview of the progress in crop lncRNA research may be relevant to future investigations on lncRNA mechanisms and their potential applications for crop improvement.

## 2 Progress in the research on lncRNAs in common crops

**Figure 1:**
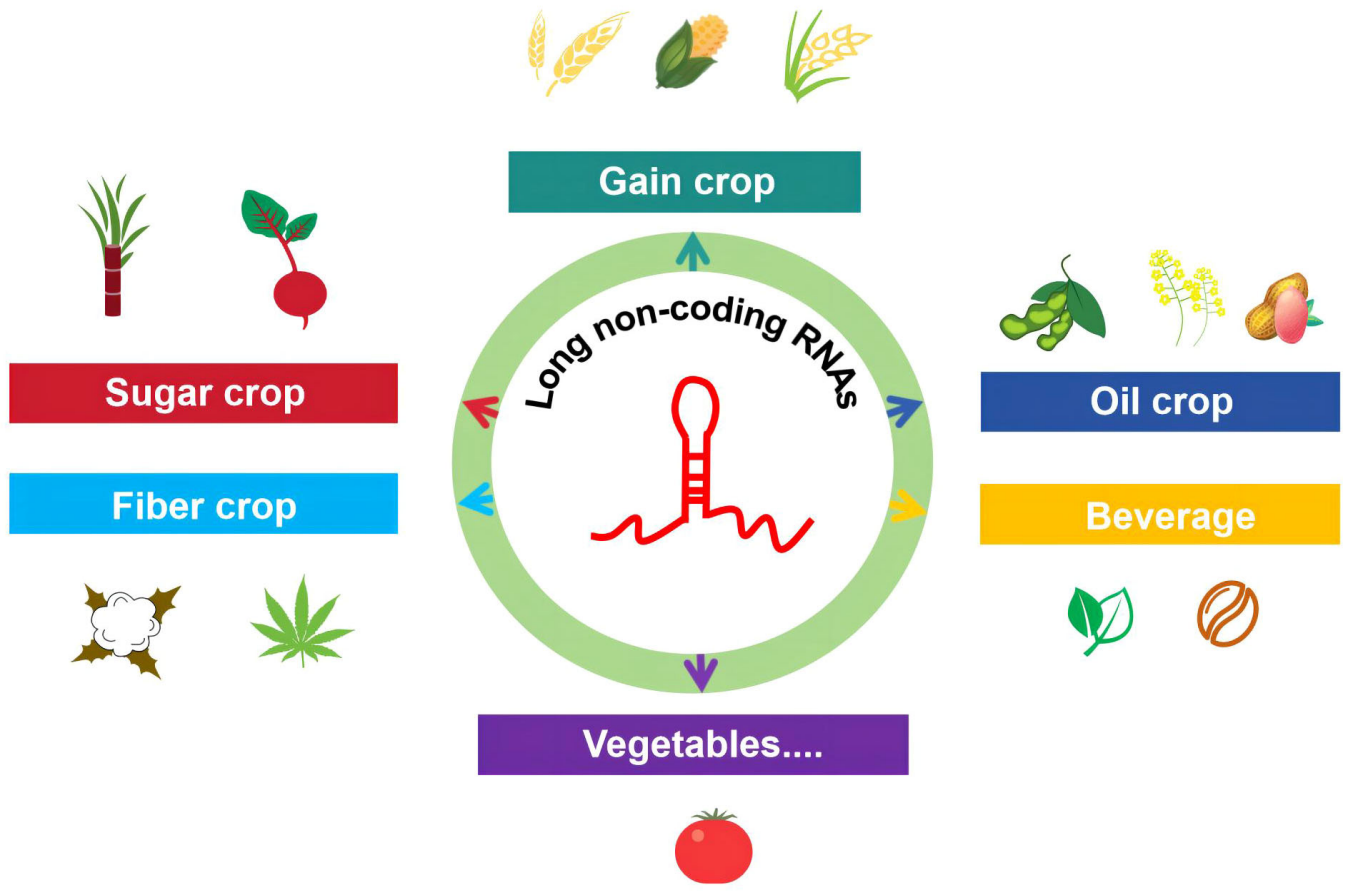
Progress in the research on lncRNAs in common crop species. This review comprehensively summarizes the functions of lncRNAs in major food crops (wheat, corn, and rice), oil crops (soybean, peanut, and rapeseed), sugar crops (sugarcane and beet), fiber crops (cotton and hemp), beverage crops (tea and coffee), and vegetables (tomato).

lncRNAs play a crucial role in regulating many biological processes in crops. Crops can be classified in different ways, one of which is the botanical classification method used in agriculture. However, since the same crop often serves multiple purposes, it is generally divided based on its primary use. Here, we introduce the research progress of lncRNA by dividing common crops into grain crops (wheat, corn, and rice), oil crops (soybean, peanut, and rapeseed), sugar crops (sugarcane and beet), fiber crops (cotton and hemp), beverage crops (tea and coffee), and vegetables (tomato) (**Figure 1**).

### 2.1 Grain crops

**Figure 2:**
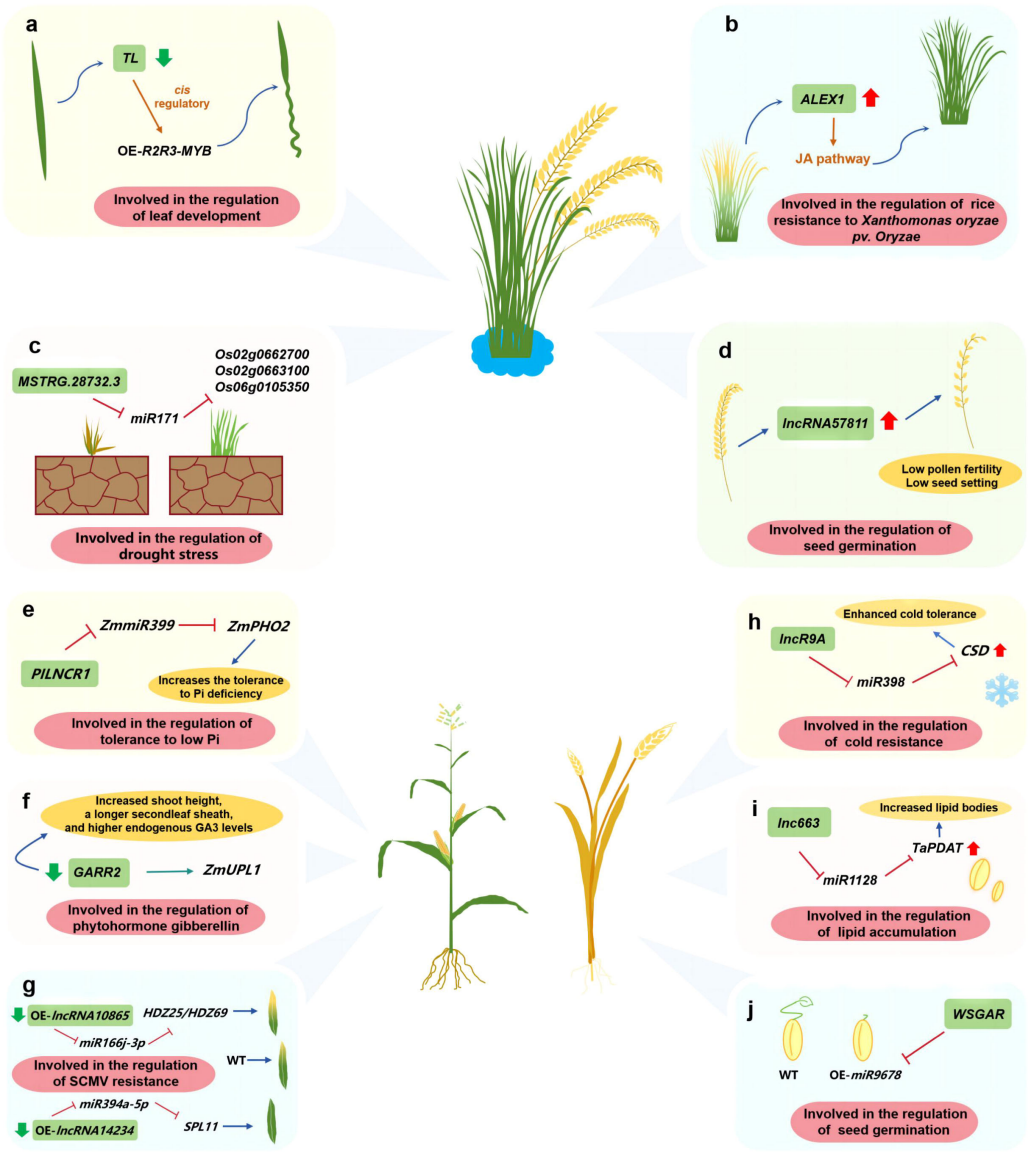
Investigating the function of lncRNAs in gain crops such as maize, rice, and wheat. **(A)** lncRNA involved in the regulation of leaf development; **(B)** lncRNA involved in the regulation of rice resistance to Xanthomonas oryzae pv. Oryzae; **(C)** lncRNA involved in the regulation of drought stress; **(D)** lncRNA involved in the regulation of seed germination; **(E)** Involved in the regulation of tolerance to low Pi; **(F)** lncRNA involved in the regulation of phytohormone gibberellin; **(G)** lncRNA involved in the regulation of SCMV resistance; **(H)** lncRNA involved in the regulation of cold resistance; **(I)** lncRNA involved in the regulation of lipid accumulation; **(J)** lncRNA involved in the regulation of seed germination.

In rice, *TWISTEDLEAF* (*TL*), is transcribed from the opposite strand of the R2R3 MYB transcription factor gene locus (*OsMYB60*). Silencing *TL* via RNA interference reportedly results in abnormal leaves (Liu et al., 2018) (**Figure 2A**). In terms of disease resistance-related lncRNAs, an RNA sequencing-based analysis of rice leaves infected with *Xanthomonas oryzae* pv. *oryzae* (*Xoo*) revealed the interactions between 39 jasmonate (JA)-related protein-coding genes and 73 lncRNAs. The overexpression of *ALEX1* enhances the resistance to *Xoo* and activates JA signaling (Yu et al., 2020) (**Figure 2B**). Research on anther and ovary meiosis in autotetraploid rice showed *lncRNA57811* overexpression significantly decreases fertility and the seed setting rate, which reflects the critical roles of lncRNAs affecting polyploid rice pollen development (Li X. et al., 2020) (**Figure 2C**). In addition, *MSTRG.28732.3*, which is a lncRNA associated with drought resistance, interacts with *miR171* to modulate the chlorophyll biosynthesis pathway, thereby influencing drought resistance through *Os02g0662700*, *Os02g0663100*, and *Os06g0105350* in rice (Yang et al., 2022) (**Figure 2D**).

In maize, lncRNAs contribute to several growth and developmental processes. For example, Pi-deficiency-induced *long-noncoding RNA1* (*PILNCR1*), which was identified following an analysis of strand-specific RNA libraries, can inhibit ZmmiR399-guided cleavage of *ZmPHO2*, ultimately affecting the ability of maize to tolerate low-Pi conditions (Du et al., 2018) (**Figure 2E**). The CRISPR/Cas9-based editing of the lncRNA *GARR2* in the GARR2KO line leads to increases in bud height, second leaf sheath length, and endogenous GA3 levels. Additionally, according to RNA pull-down assays, *GARR2* can influence the abundance of its target (*ZmUPL1*) during the gibberellin (GA) response (Li W. et al., 2022) (**Figure 2F**). Furthermore, sugarcane mosaic virus (SCMV)-responsive lncRNA–miRNA–mRNA networks have been established. The lncRNA10865-miR166j-3p-HDZ25/HDZ69 and lncRNA14234-miR394a-5p-SPL11 modules played roles in maize resistance to SCMV infection. Among them, after *lncRNA10865* and *lncRNA14234* were silenced, SCMV symptoms were aggravated and alleviated, respectively (Gao et al., 2023) (**Figure 2G**).

In a previous study on the mechanism mediating the cold resistance of winter wheat, *lncR9A* was revealed to function as a competing endogenous RNA (ceRNA) that regulates the cooperative interaction between *tae-miR398* and *TaCSD1* under cold conditions (Lu et al., 2020) (**Figure 2H**). An investigation on wheat grain fat biosynthesis detected a lncRNA that serves as a ceRNA modulating lipid accumulation through *TaPDAT*. More specifically, on the basis of a GFP reporter assay, *lnc663* can sequester *miR1128* through complementary interactions to up-regulate *TaPDAT* expression in tobacco (Madhawan et al., 2023) (**Figure 2I**). In addition, lncRNAs may also regulate abscisic acid/GA signaling to affect seed germination. The overexpression of *miR9678* delays wheat seed germination by decreasing the bioactive GA content. Interestingly, *miR9678* targeted the lncRNA *WSGAR* (Guo et al., 2018) (**Figure 2J**).

In summary, lncRNAs modulate the growth, development, and biotic and abiotic stress responses of major grain crops, including rice, maize, and wheat. While these biological processes may affect grain crop yields, the molecular mechanisms underlying lncRNA functions in grain crops must be more precisely deciphered to improve grain crop production.

### 2.2 Oil crops

**Figure 3:**
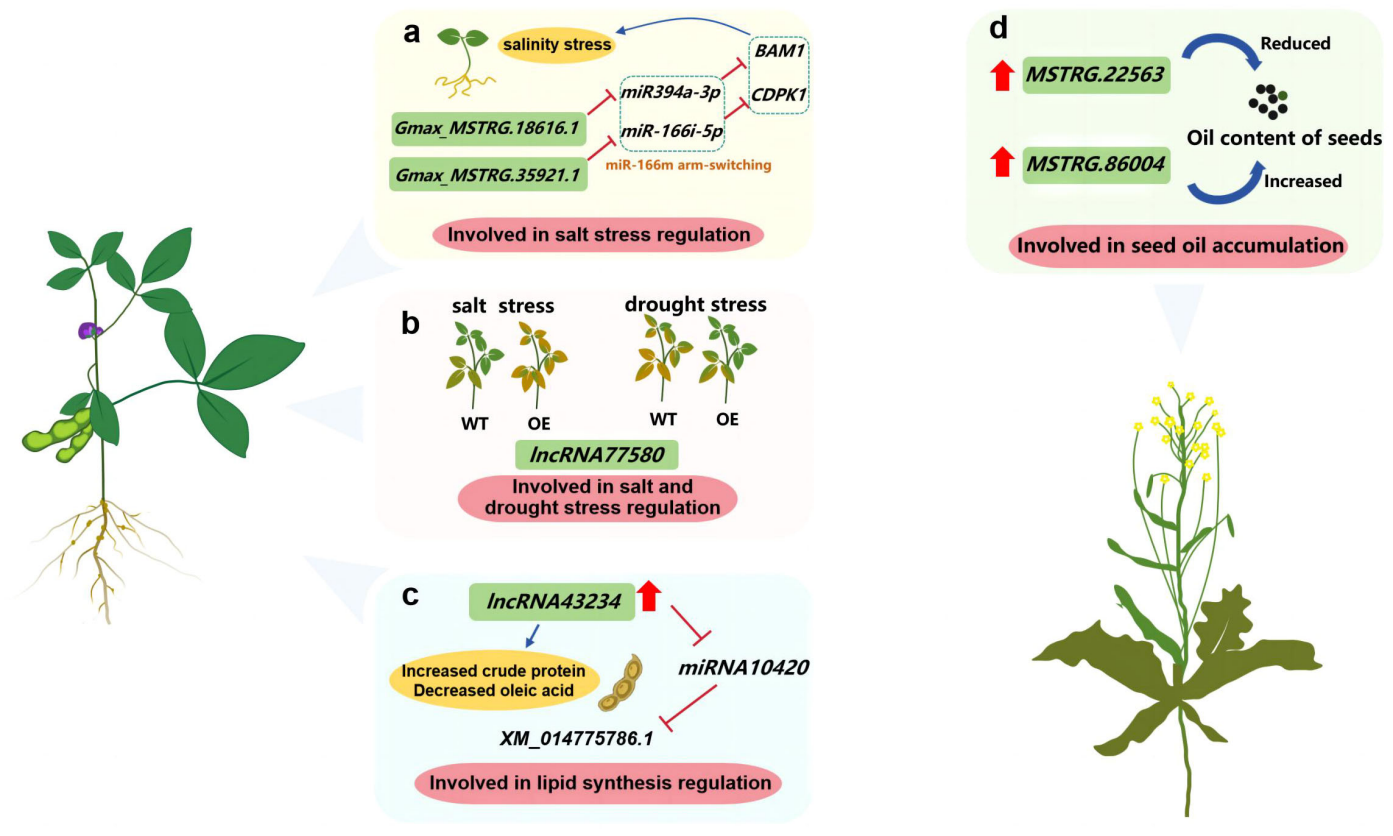
Investigating the function of lncRNAs in oil crops such as soybean and rapeseed. **(A)** lncRNA involved in salt stress regulation; **(B)** lncRNA involved in salt and drought stress regulation; **(C)** lncRNA involved in lipid synthesis regulation; **(D)** lncRNA involved in seed oil accumulation.

Soybean is one of the main oil crops. In the study of soybean salt response stress, the interaction between *Gmax_MSTRG.35921.1* and *miR166i* was verified by LAMP assay followed by RT-PCR, which indicated the potential regulatory role of lncRNA under salinity stress (Li C. et al., 2022) (**Figure 3A**). In addition, overexpressing *lncRNA77580* in soybean could increase the drought tolerance and seed yield by increasing the number of seeds per plant (Chen X. et al., 2023) (**Figure 3B**). The lncRNA43234-miRNA10420-XM_014775781.1 network related to lipid synthesis was screened out by full-length transcriptome sequencing for Wild type (WT) soybean “JN18” (Jishendou 2006) and low linolenic acid mutant “MT72”. Overexpression of *lncRNA43234* resulted in increased protein content and decreased oleic acid content in *Arabidopsis thaliana* seeds (Ma et al., 2021; Zhang A. et al., 2023) (**Figure 3C**).

*Brassica napus* L., which is one of three types of oilseed rape, has the highest grain yield among all oilseed rape varieties. The seed oil content decreases by 3.1%–3.9% following the overexpression of *MSTRG.22563*, but increases by approximately 2% if *MSTRG.86004* is overexpressed (Li Y. et al., 2023) (**Figure 3D**). However, clubroot disease causes significant *Brassica* yield losses. A total of 464 differentially expressed lncRNAs were identified in the roots of resistant plants challenged with *Plasmodiophora brassicae*, with most of the genes targeted by these lncRNAs associated with plant–pathogen interactions and hormone signaling pathways (Summanwar et al., 2021). Furthermore, the positive effects of lncRNAs on *B. napus* drought tolerance have been elucidated. Certain lncRNAs affecting plant hormone signaling and defense mechanisms are co-expressed with protein-coding genes (Tan et al., 2020).

In 2019, a weighted correlation network analysis established a co-expression network comprising 4,713 lncRNAs, which enabled the identification of lncRNAs associated with the growth and development of various peanut tissues (Zhao et al., 2019). Concurrently, seeds from two peanut recombinant inbred lines (RIL8) with differing seed sizes were subjected to strand-specific whole transcriptome sequencing at 15 and 35 days after flowering (DAF). An examination of differentially expressed genes and qPCR data revealed the importance of 11 lncRNAs and their cis-acting target genes for peanut seed development (Ma et al., 2020). Furthermore, 10 lncRNAs functioned as ceRNAs involved in oxidation–reduction processes and other metabolic pathways during a root-knot nematode infection of peanut (Xu et al., 2022).

The findings of previous studies indicate lncRNAs modulate the oil content and quality of oil crops (e.g., soybean and rapeseed). Clarifying the gene regulatory network governing lipid metabolism is crucial for enhancing oil crop yield and quality. A thorough examination of the key lncRNAs associated with oil metabolism will provide relevant insights into the molecular mechanisms underlying lipid metabolism in oil crops.

### 2.3 Sugar crops

The main sugar crops include sugarcane (*Saccharum officinarum* L.) and sugar beet (*Beta vulgaris*). In a study exploring sugarcane tiller development, 310 conserved lncRNAs were screened on the basis of a PacBio Iso-Seq analysis of leaf and tiller bud samples (Yan et al., 2021). Previous studies had shown that *miR408* is important for the interaction between sugarcane and microorganisms. A long intergenic noncoding RNA (lincRNA) with significant complementarity to *miR408* was predicted to act as miRNA bait, with inhibitory effects on the regulation of canonical miR408 targets (Thiebaut et al., 2017). Other studies showed that miRNAs influence sugarcane growth and development, stress resistance, and other processes (Li A. M. et al., 2023; Gao et al., 2022). The research conducted to date on sugarcane lncRNAs has primarily relied on predictions, which will need to be experimentally verified. In particular, the regulatory effects of lncRNAs on sweetness-related genes should be characterized.

Changes in gene expression during sugar beet responses to salt stress have been elucidated via whole transcriptome RNA-seq and degradome sequencing analyses, which identified 61 differentially expressed lncRNAs in roots and 55 target genes (Li J. et al., 2020). In another study, sugar beet responses to drought stress were examined, resulting in the detection of 386 differentially expressed lncRNAs; the expression of the most significantly up-regulated lncRNA increased more than 6,000-fold, whereas the expression of the most significantly down-regulated lncRNA decreased more than 18,000-fold (Zou et al., 2023). In sugar beet, the gene (*Bv8_189980_mizi.t1*) targeted by the lncRNA *MSTRG.26204.1* encodes a B3 domain-containing transcriptional repressor (VAL1-like), suggesting this gene may be associated with vernalization. Hence, lncRNAs may be involved in the sugar beet vernalization process (Liang et al., 2022).

Although there is evidence indicating lncRNAs affect the drought resistance as well as the growth and development of sugar crops, their contribution to sugar biosynthesis and the underlying molecular mechanism remain unclear. Exploring the effects of lncRNAs on plant sugar biosynthesis pathways may provide insights relevant to regulating key sugar crop traits.

### 2.4 Beverage crops

Cocoa, coffee, and tea are the main beverage crops worldwide. To date, there has been limited research on cocoa lncRNAs, but coffee and tea lncRNAs have been identified and functionally characterized. In *Coffea canephora*, 2,384 high-confidence lncRNAs were identified on the basis of a comprehensive genome-wide analysis (Lemos et al., 2020). A total of 10,564 lncRNAs were identified in another coffee species (*Coffea arabica* L.). Their involvement in important biological processes was predicted by a Gene Ontology (GO) analysis (Abdel-Salam et al., 2021). In tea (*Camellia sinensis*), lncRNAs are involved in disease resistance-related mechanisms. Additionally, in *C. sinensis* ‘Baiye No. 1’, differentially expressed lncRNAs participate in responses to periodic albinism through the GAMYB–miR159–lncRNA regulatory network (Xu et al., 2023). A recent study indicated *MSTRG.20036*, *MSTRG.3843*, *MSTRG.26132*, and *MSTRG.56701* influence the development of tea leaf spot disease through cis-regulatory mechanisms (Huang et al., 2023). Another lncRNA (*MSTRG.139242.1*) may modulate the response to salt stress through Ca^2+^ ATPase 13 in the Ca^2+^ transport pathway (Wan et al., 2020). In response to daylight-induced withering, lncRNAs alter flavonoid and terpenoid metabolic pathways as well as JA/methyl jasmonate biosynthesis and signal transduction in oolong tea (*C. sinensis*) (Zhu et al., 2019). Thus, lncRNAs help regulate disease resistance mechanisms and salt stress responses in beverage crops. They also regulate the production of biologically active substances that influence the flavor profile and other characteristics of beverage crops.

### 2.5 Fiber crops

**Figure 4:**
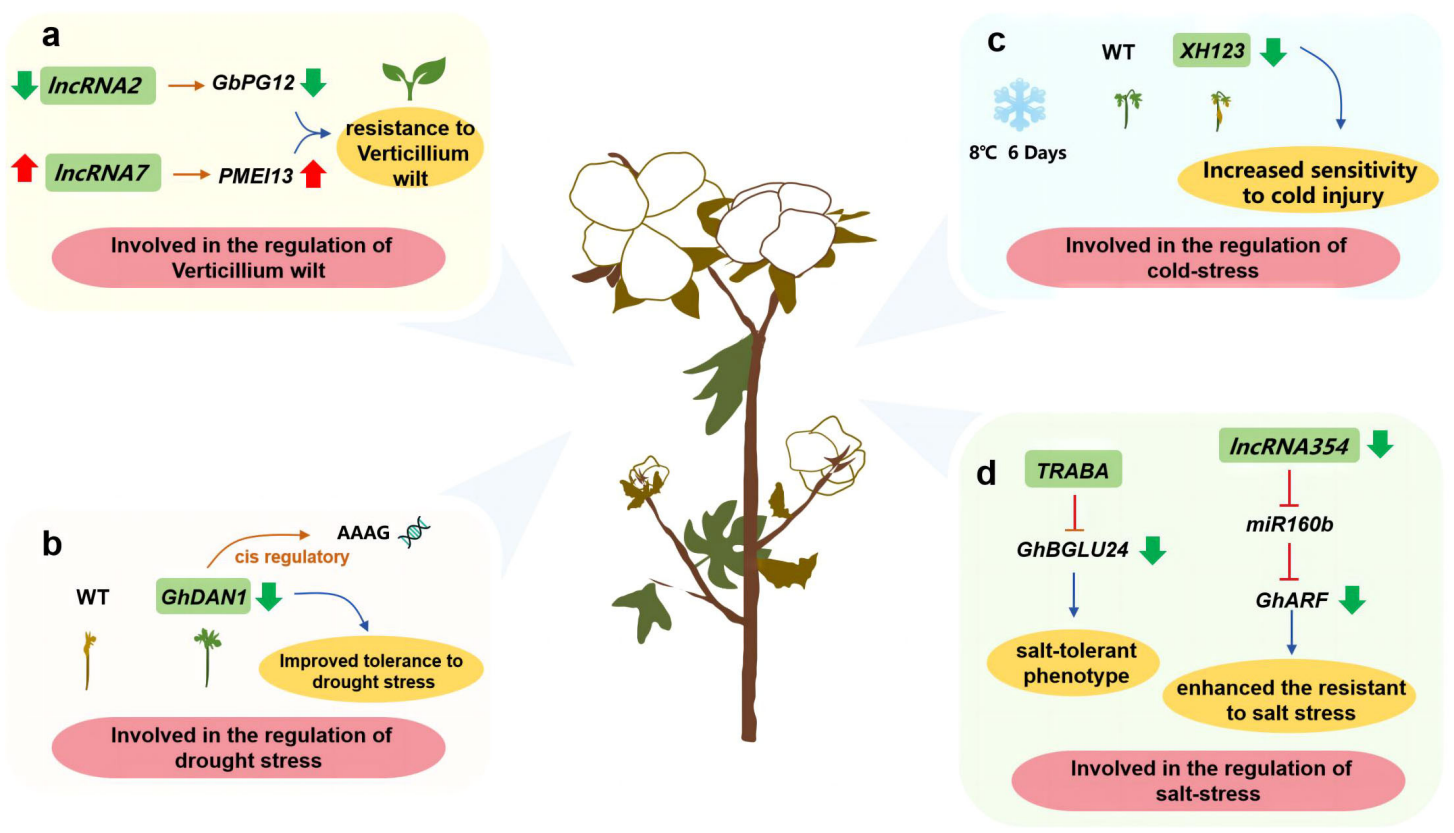
Investigating the function of lncRNAs in fiber crops such as cotton. **(A)** lncRNA involved in the regulation of Verticillium wilt; **(B)** lncRNA involved in the regulation of drought stress; **(C)** lncRNA involved in the regulation of cold-stress; **(D)** lncRNA involved in the regulation of salt-stress.

Cotton seeds produce fiber. Some studies have shown that lncRNAs are involved in the disease resistance of cotton. For example, *lncRNA2* and its target gene *PG12* negatively regulate cotton resistance to verticillium wilt, while *lncRNA7* and its target gene *PMEI13* have the opposite effect (Zhang L. et al., 2022) (**Figure 4A**). Interestingly, lncRNAs are also involved in cotton responses to abiotic stress. More specifically, *DAN1*, which is a lincRNA associated with drought responses, can regulate AAAG motif-containing genes in the auxin response pathway (Tao et al., 2021) (**Figure 4B**). Another lincRNA, *XH123*, was revealed to control the cold stress response of cotton seedlings (Cao et al., 2021) (**Figure 4C**). The salt-responsive lncRNAs *TRABA* and *lncRNA354* serve as upstream regulators that control the expression of the salt stress response-related genes *GhBGLU24* and *GhARF*, respectively (Zhang X. et al., 2021; Cui et al., 2024) (**Figure 4D**). In cotton, *MSTRG 2723.1* mediates the expression of key genes related to fatty acid metabolism, the MYB25-mediated pathway, and pectin metabolism to regulate fiber synthesis (Zou et al., 2022). In addition to cotton, hemp is another major fiber crop. In ramie (*Boehmeria nivea* L. Gaud), a MYB gene (*BntWG10016451*) is targeted by *lncRNA00022274*. The overexpression of this gene reportedly increases fiber production in *A. thaliana* (Fu et al., 2023). Considered together, the findings of earlier studies suggest lncRNAs play crucial roles in fiber crop responses to biotic and abiotic stresses, while also influencing fiber formation. These investigations have increased our understanding of how lncRNAs regulate plant fiber development.

### 2.6 Vegetables

**Figure 5:**
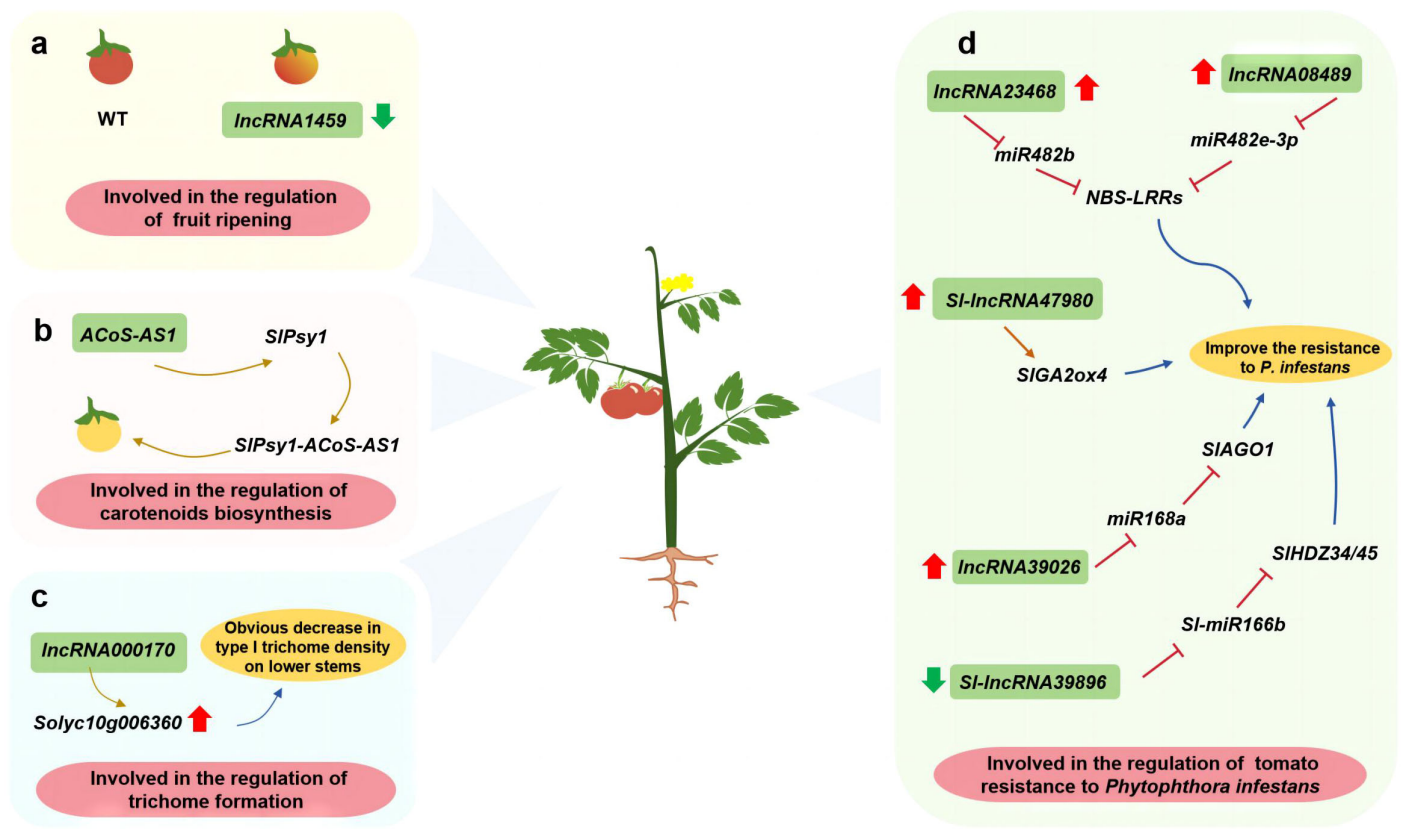
Investigating the function of lncRNAs in vegetable such as tomato. **(A)** lncRNA involved in the regulation of fruit ripening; **(B)** lncRNA involved in the regulation of carotenoids biosynthesis; **(C)** lncRNA involved in the regulation of trichome formation; **(D)** lncRNA involved in the regulation of tomato resistance to Phytophthora infestans.

Tomato (*Solanum lycopersicum* L.) is one of the most important vegetable crops. The silencing of *lncRNA1459* reportedly decreases ethylene accumulation and carotenoid biosynthesis in tomato, with detrimental effects on fruit ripening (Li et al., 2018) (**Figure 5A**). During carotenoid biosynthesis, octahydro-lycopene synthase (PSY) catalyzes the formation of two GGPP molecules. Additionally, trans-splicing between *SlPsy1* and the lncRNA *ACoS-AS1* leads to the formation of yellow tomato fruit (Xiao et al., 2020) (**Figure 5B**). Overexpressing *Solyc10g006360* decreases the formation of type I trichomes. An earlier study showed *lncRNA000170*, which is transcribed from the complementary strand of *Solyc10g006360*, may affect multicellular trichome formation by inducing target gene expression (Liao et al., 2020) (**Figure 5C**). In the study of tomato against *Phytophthora infestans*, overexpression of *Sl-lncRNA47980* up-regulated the expression of *SlGA2ox4*, while overexpression of *lncRNA39026* down-regulated the expression of *miR168a* and increases the expression of *SlAGO1*. In tomato, the overexpression of *lncRNA23468* and *lncRNA08489* significantly decreases the expression of *miR482b* and *miR482e-3p*, respectively, but the expression of target genes encoding NBS-LRR proteins increases significantly. These lncRNAs positively regulate the resistance of tomato plants to *P. infestans*. Conversely, *Sl-lncRNA39896* negatively regulates tomato resistance to *P. infestans*; this lncRNA functions as an endogenous target mimic of *Sl-miR166b* that controls *HDZ* expression (Jiang et al., 2019; Hou et al., 2020; Su et al., 2023; Liu et al., 2022; Hong et al., 2022) (**Figure 5D**).

## 3 Main research strategies for crop lncRNA

**Figure 6:**
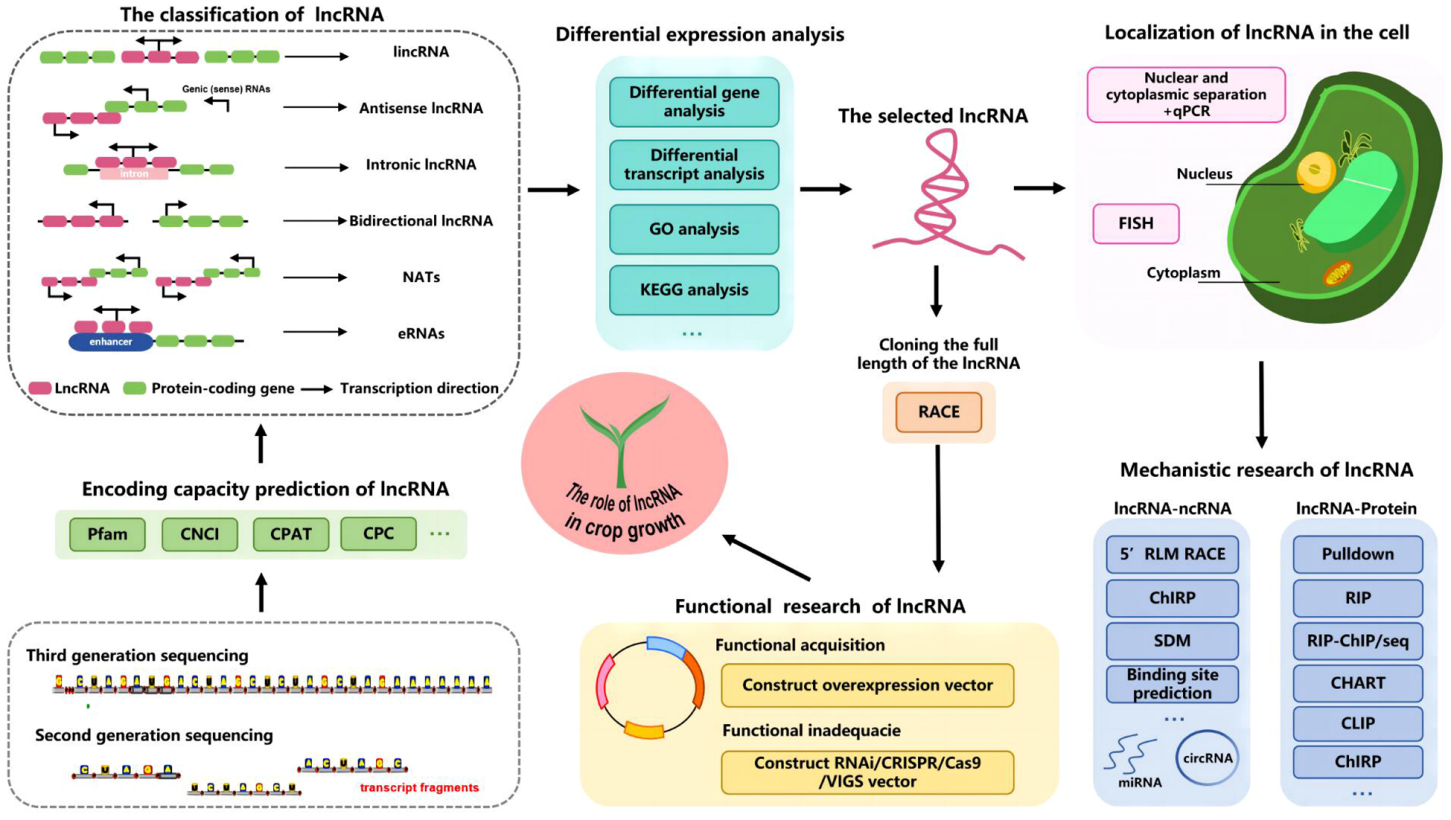
Flow chart of the research strategy for identifying lncRNAs in crops.

In this section, we highlight the primary methods used to investigate lncRNAs in common crops (**Figure 6**). Novel lncRNAs are generally identified in studies involving high-throughput sequencing (e.g., after various treatments, at selected time points, or in specific tissues) followed by transcript splicing and assembly. The expression levels of candidate lncRNAs and mRNAs are then analyzed to screen for differential expression. Plant studies focused on lncRNA functions mainly involve the application of second-generation sequencing technologies, despite the increasing popularity of third-generation sequencing. Although third-generation sequencing technology may be better than earlier sequencing technologies for sequencing genomes and transcriptomes, its widespread application may be restricted by its high costs. Because of its advantages (e.g., short reads, high throughput, and high accuracy), second-generation sequencing technology is still commonly used for plant research. However, third-generation full-length transcriptome sequencing has generated high-quality complete transcriptomes (Zhu et al., 2013; Rhoads and Au, 2015; van Dijk et al., 2023).

Sequenced transcripts may be screened for lncRNAs using diverse methods (e.g., CPC, CNCI, CPAT, and pfam protein domain analysis) (Zhang J. et al., 2022; Liu X. Q. et al., 2019; Duan Y. et al., 2020). The PmlIPM model was recently used to predict plant miRNA–lncRNA associations (Tang and Ji, 2023). By integrating a paired sgRNA design with an off-target analysis, CRISPRlnc can be used to design CRISPR/Cas9 sgRNAs for ncRNAs (Yang et al., 2024). The identified lncRNAs, including lincRNAs, intronic lncRNAs, antisense RNAs, NATs, bidirectional lncRNAs, and eRNAs, may be classified according to their genomic locations relative to protein-coding genes. This classification is useful for future studies on lncRNA functions (Chekanova, 2015; Wierzbicki et al., 2021). The expression and functional significance of lncRNAs and protein-coding genes must be clarified. Differentially expressed genes and consistently highly expressed genes may play crucial roles in key metabolic pathways and biological processes. Several databases (e.g., KEGG, GO, GreeNC, PlncRNADB, LncTar, NONCODE, and other lncRNA-related databases) have been used to predict lncRNA functions and select lncRNAs for further analyses (Di Marsico et al., 2022; Li et al., 2015; Quek et al., 2015; He et al., 2008).

Full-length lncRNAs may be amplified via rapid amplification of cDNA ends (RACE) for in-depth analyses when only transcript fragments are available (Zhou et al., 2023). The mechanisms mediating the regulatory effects of lncRNAs in plants vary because of the diversity in the cellular locations of lncRNAs. The localization of lncRNAs in cells can be determined by conducting a qPCR analysis of lncRNA expression in the isolated nucleus and cytoplasm or a fluorescence *in situ* hybridization assay (Qin and Xiong, 2019; Yang et al., 2020). Nuclear lncRNAs interact with DNA, RNA, proteins, and other molecules to regulate chromosome structure and function, while also controlling gene transcription (cis- or trans-regulation). In contrast, cytoplasm-localized lncRNAs have post-transcriptional regulatory effects (Zhang M. et al., 2021; Dietrich et al., 2015; Hu et al., 2023). The interaction between lncRNAs and proteins may be confirmed using various approaches, including pull-down assays, RNA-binding protein immunoprecipitation (RIP), cross-linking immunoprecipitation (CLIP), and chromatin isolation by RNA purification (ChIRP) (Ferrè et al., 2016; Jiang et al., 2023). The ceRNA mechanism is currently a major topic of interest among researchers. Various methods, including 5′ RLM RACE, ChIRP, and binding site prediction, are useful for investigating the interaction between lncRNAs and miRNAs or circRNAs (Zhang L. et al., 2023; Rao et al., 2022). After the initial verification, lncRNA functionality must be confirmed. This involves constructing overexpression vectors that are subsequently inserted into plants for an analysis of the effects of lncRNA overexpression. Additionally, RNAi, CRISPR/Cas9, VIGS, and other technologies were utilized to suppress target genes or induce mutations, ultimately confirming the function of the target lncRNA (Aydinoglu and Kuloglu, 2023; Bravo-Vázquez et al., 2023).

## 4 Discussion and prospects

In addition to conventional breeding techniques, technological advances (e.g., transgenic technology) have resulted in several alternative methods for improving crop traits (Kumar et al., 2020). Third-generation sequencing technologies have facilitated the detection and characterization of functional genes beyond protein-coding genes, with the identified lncRNAs potentially useful for enhancing crop traits (Xu et al., 2020; Chen T. et al., 2023). We herein reviewed the effects of lncRNAs on the growth and development of key crops. Crops have been classified according to their uses as well as their botanical characteristics. Field crops are frequently divided into three categories: edible crops, industrial raw materials, and feed crops. However, because of the multifunctionality of many crops, in this review, we classified them according to their primary use. We specifically focused on lncRNAs with confirmed regulatory functions in crops, rather than those that are merely predicted to be associated with crop growth and development. We also summarized the major findings of studies on lncRNA functions in various plant species. The importance of lncRNAs for regulating crop growth was emphasized (**Table 1**).

**Table 1:** Functions of lncRNAs in other plants.

Gene Name	Origin	Mechanism	Gene function	Research significance	References
*SEAIRa*	*Arabidopsis*	Represses *SE* expression	Turn led to serrated leaves	Uncover an epigenetic mechanism mediated by the lncRNA *SEAIRa* that modulates *SE* expression	(Chen W. et al., 2023)
*T5120*	*Arabidopsis*	Interacts with *NLP7* and *NRT1.1*	Regulate nitrate signalling	Reveal a new regulatory mechanism in which lncRNA T5120 functions in nitrate regulation, providing new insights into the nitrate signalling network	(Liu F. et al., 2019)
*FLAIL*	*Arabidopsis*	As a trans-acting RNA molecule	Affect alternative splicing and represses flowering	Suggest lncRNAs as accessory components of the spliceosome that regulate AS and gene expression to impact organismal development	(Jin et al., 2023)
*PMAT-PtoMYB46*	*Populus*	Represses *PtoMATE* and *PtoARF2*	Promote Pb^2+^ uptake and plant growth	Demonstrate the involvement of lncRNAs in response to Pb^2+^ in poplar	(Chen et al., 2022)
*lncRNATCONS00065739*	*Ammopiptanthus nanus*	As an endogenous competitive target of *miR530*	Contribute to the cold stress adaptation	Provide new data for understanding the biological roles of lncRNAs in response to cold stress in plants	(Zhu et al., 2023)
*HILinc1*	*Pyrus* spp.	Facilitates *PbHSFA1b* through stabilizing *PbHILT1* transcripts	Enhance pear thermotolerance	Investigate the role of lncRNA in enhancing heat tolerance in pears and offer suggestions for enhancing both yield and quality	(Zhang Y. et al., 2022)
*DglncTCP1*	*Chrysanthemum morifolium* Ramat.	Cis-regulatory role	Play a key role in improving the cold tolerance of chrysanthemum	Suggest that natural antisense lncRNA plays a key role in improving the cold tolerance of chrysanthemum	(Li X. et al., 2022)
*MSTRG.85814*	*Malus domestica*	Cis-regulatory role	Activate proton extrusion involved in the Fe-deficiency response	Reveal a mechanism by which lncRNA promotes environmental Fe-deficiency stress adaption	(Sun et al., 2020)
*FRILAIR*	Strawberry	Act as a noncanonical target mimic of *miR397*	Modulate strawberry fruit ripening process	Characterize a functional model for lncRNA-miRNA-gene regulation in the regulation of strawberry fruit ripening	(Tang et al., 2021)

Research on lncRNAs in crops lags behind the corresponding research in animals. Hence, there are numerous gaps in our knowledge that will need to be addressed. Nevertheless, numerous functional lncRNAs had been identified and functionally annotated in various model plants (Jampala et al., 2021). Further research is needed to elucidate the functions of lncRNAs in crop species as well as the underlying mechanisms. Unlike the extensively annotated protein-coding genes, lncRNAs are frequently inadequately annotated. Crop lncRNAs may be annotated and classified using the methods that were employed for annotating animal lncRNAs (Ballarino et al., 2023; Park and Kim, 2023). However, in addition to RNA-seq technology, animal lncRNAs can be identified using gene chip technology. Although gene chips are widely used for animal and pharmaceutical research, they are too expensive for most agricultural scientific research institutions. Therefore, the application of gene chip technology for annotating plant lncRNAs may depend on a decrease in the associated costs (Morohashi et al., 2009; Verma et al., 2022; Song et al., 2023). The precise genome locations and functional significance of numerous lncRNAs remain unknown. The biological functions of lncRNAs are intricately linked to their secondary structure. Unfortunately, existing programs and tools for lncRNAs often prioritize the complete secondary structure, while overlooking local structures crucial for biological functions (Herman et al., 2022; Sanbonmatsu, 2022). To further annotate lncRNAs, their secondary structures will need to be explored at a higher resolution. New sources of lncRNAs were continually being identified and classified (Chorostecki et al., 2023; Li C. et al., 2023; Mattick et al., 2023).

Further advances in related technologies may lead to a more comprehensive elucidation of lncRNA functions and the associated mechanisms. The development of more efficient programs and tools has enabled researchers to acquire increasingly accurate insights into lncRNAs in crops (Sheng et al., 2023). Moreover, CRISPR technology, which was initially used for plant genome editing in 2013, has been exploited to improve crop traits. Progress in the related research has resulted in enhanced breeding practices, but it has also simplified the classification of lncRNA functions, thereby enabling researchers to functionally validate lncRNAs in crops (Atia et al., 2024; Chovatiya et al., 2024). In this context, lncRNAs are also expected to play a more essential role in the genetic breeding of crops, the development of biological resource, the engineering of plant cells, and other areas. Improved living standards, farming system changes, research on plant diseases and pest infestations, and the development of specialized crops have necessitated the generation of new crop varieties. Furthermore, varietal replacement rates have increased. Hence, transgenic breeding can no longer be reserved for exploring protein-coding genes. Functional lncRNAs will need to be identified and analyzed regarding their utility for promoting crop production. This may increase crop yields, enhance crop stress resistance, and optimize the contents of beneficial substances, thereby increasing the efficiency of agricultural production (Palos et al., 2023; Yang et al., 2023). Furthermore, lncRNAs may be considered as key factors influencing cellular architecture. By culturing and proliferating cells or modifying specific plant cell characteristics, breeders can generate economically valuable crop products (Gonzales et al., 2024). Although research on crop lncRNAs is in its nascent stages, studies conducted to date have highlighted the importance of lncRNAs as well as the need for additional research to more precisely determine their roles in crops.

## Author contributions

AZ: Writing – original draft. WP: Writing – review & editing. YW: Writing – review & editing. YL: Writing – review & editing. JW: Writing – review & editing. SL: Writing – review & editing. XC: Writing – review & editing. HL: Writing – original draft. DY: Writing – original draft. RZ: Writing – original draft.
